# The Use of Honey for Cicatrization and Pain Control of Obstetric Wounds: A Systematic Review and Meta-Analysis of Randomized Controlled Trials

**DOI:** 10.3390/nu16020185

**Published:** 2024-01-05

**Authors:** Bárbara Ferraz Barbosa, Francisco Cezar Aquino de Moraes, Bruno Araujo Alves da Silva, Camila Bordignon Barbosa, Izael Pereira da Silva, Emanuele Rocha da Silva, Jamile Cristine Marques Barros, Laissa Wane Cavalcante Rebouças, Ney Pereira Carneiro dos Santos, Marianne Rodrigues Fernandes

**Affiliations:** 1 Department of Medicine, University of Aquino Bolivia, Santa Cruz de la Sierra 0701, SC, Bolivia; bferraz.barbosa@hotmail.com (B.F.B.); bordignoncamila4@gmail.com (C.B.B.); 2 Oncology Research Center, Federal University of Pará, Belém 66073-005, PA, Brazil; emanueleersilva@gmail.com (E.R.d.S.); jamilecbarros@gmail.com (J.C.M.B.); npcsantos.ufpa@gmail.com (N.P.C.d.S.); fernandesmr@yahoo.com.br (M.R.F.); 3 Department of Medicine, State University of Ceará, Fortaleza 60714-903, CE, Brazil; bru.araujo@aluno.uece.br; 4 Department of Medicine, Federal University of Amazonas, Manaus 69020-160, AM, Brazil; izaelbatista999@gmail.com; 5 Gynecology and Obstetrics Service of Clinical Hospital of Ribeirão Preto, University of São Paulo, Ribeirão Preto 14049-900, SP, Brazil; lwcreboucas@hcrp.usp.br

**Keywords:** honey, episiotomy, cesarean section, wound healing, pain

## Abstract

Objective: Several studies point to antibacterial properties and beneficial effects of honey on scar tissue formation, which is a low-cost and easy-to-use option. This study aimed to compare honey versus a placebo for cicatrization and pain control of obstetric wounds, and determine if one is superior to the other, in terms of efficacy, through a meta-analysis. Methods: We searched PubMed, Scopus, Cochrane Central Register of Controlled Trials, and Web of Science. Two independent investigators identified randomized controlled trials (RCTs) comparing honey and a placebo for obstetric wounds. The primary outcomes were wound healing and pain control. Results: Five randomized controlled trials and 353 patients were included, of whom, 177 (50.1%) were treated with honey. Differences were not found in the final wound healing between the honey and placebo groups (MD −0.34; 95% CI −1.13, 0.44; *p* = 0.39); however, there was a decrease in pain levels in the middle of the treatment (SMD −0.54; 95% CI 0.83 to 0.25, *p* = 0.03), reduction in the use of pain medication (ORR 0.26; 95% CI 0.08, 0.86; *p* = 0.03), increase in personal satisfaction in women who underwent the intervention (ORR 0.81; 95% CI 0.65, 0.98), and reduction in complications. Conclusion: According to the study results, honey treatments showed greater efficiency and provided benefits to the patients by accelerating wound healing and decreasing reported pain.

## 1. Introduction

The moment of childbirth, whether normal or through a surgical procedure, can lead to complications. This highlights the need for effective treatments and proper wound management using cost-effective methods that promote maternal well-being, reduce mortality, improve healing, and control pain [1,2].

Complications arising from normal delivery, such as various types of perineal tears, as well as those resulting from interventions (episiotomy and caesarean section), are associated with increased physical and mental stress caused mainly by pain, which can affect the development of the mother–newborn bond, especially without proper treatment and management [1,3]. Some classes of medication are routinely used in clinical practice to relieve pain; however, these medications have drawbacks, as they may not only prolong the healing process but also cause undesirable side effects [4].

Honey is a natural product that has recently been reintroduced into medical practice, but its use in wound healing goes back centuries and has its origins in ancient civilizations. Studies have shown that honey’s healing properties are due to its acidity and hydrogen peroxide content, in addition to its osmolarity; its composition is rich in sugars, vitamins, minerals, flavonoids, and phenolic acids, which act as nutritional and antioxidant factors, as well as prostaglandins and nitric oxide, which play an important role in honey’s anti-inflammatory and antimicrobial powers [5,6,7]. 

The composition of honey depends mainly on the flowers, geographical region, climate, and species of bees, and can vary in quantity and quality of nutrients [6]. Medihoney and Manuka honey are two different types of honey used in the wound-healing process in different countries around the world; their properties have been studied and the results show similarities, even though the different plants result in different variations and bactericidal abilities [7].

Medical-grade honey is starting to be seen as a promising treatment option due to its broad spectrum of antimicrobial and anti-inflammatory properties, in addition to stimulating the migration of fibroblasts and the deposition of collagen in the injured area, thus promoting wound healing and regeneration [4,8]. Due to all these properties, honey has been used in various types of wounds, primarily for burns, surgical incisions, infected wounds, chronic ulcers [9,10,11,12], and in postoperative management of cesarean sections [8]. Since pain and the healing process can interfere with both a proper breastfeeding process and the creation of an early bond with the neonate, proper wound management is extremely important for maternal recovery and early childcare [1,4].

Despite previous promising studies on honey, there is still no consensus on its beneficial effects in relation to outcomes such as pain reduction and improved healing. Therefore, the aim of this study was to compare the use of honey in obstetric wounds, evaluating these clinical outcomes, the use of analgesics and antibiotics, diet used, level of satisfaction, and complications. The aim was to provide more convincing scientific evidence, in light of the weak strength of individual studies.

## 2. Materials and Methods

### 2.1. Protocol 

This meta-analysis followed the guidelines of the preferred reporting items for systematic reviews and meta-analysis (PRISMA) statement and the recommendations of the Cochrane Collaboration [13].

### 2.2. Eligibility Criteria

Inclusion in this meta-analysis was restricted to studies that met all of the following eligibility criteria: (1) randomized clinical trials only; (2) comparison of the use of honey versus a placebo and/or control group; (3) only studies published in the English language; (4) subjects were women with obstetric surgical wounds from a cesarean section or episiotomy with REEDA scale (redness, edema, ecchymosis, discharge, and approximation of wound edges) scores; (5) reported the clinical outcomes of interest. We excluded studies (1) with overlapping patient populations; (2) that were meta-analyses or literature reviews; (3) animal studies; or (4) conference abstracts or studies reported in an incomplete form (lack of standardized measures). There were no restrictions on the age, education, or occupation of the patients, number of pregnancies or deliveries, gestational time, or other exclusion criteria that were used in the individual studies.

### 2.3. Search Strategy and Data Extraction

A systematic search was conducted for studies that met the eligibility criteria and were published from “inception” through November 2022. The search strategy was based on the following search terms: ‘honey’, ‘cesarean section’, ‘episiotomy’, and ‘wound healing’; and was performed by two different authors (F.B. and S.B.) in the following databases: PubMed, Scopus, Cochrane Central Register of Controlled Trials, and Web of Science. In addition to the database search, the references for included studies were manually checked. There were language restrictions on articles, which should be published in English. Three authors (F.B., S.B., and B.C.) independently extracted baseline characteristics and outcome data following pre-defined search criteria. Disagreements were resolved through consensus among the authors.

### 2.4. Endpoints and Subgroup Analyses

The outcomes of interest were: (1) wound healing of cesarean section, episiotomy, or vaginal tears evaluated with the REEDA scale; and (2) pain intensity was assessed using visual analog scales (VAS). Definitions of wound healing did not vary between studies, and the authors used six dimensions of REEDA for redness, edema, ecchymosis, secretion, perineal approximation, and perineal tissue approximation. The lower the score, the better the wound healing. The studies also reported pain levels at baseline and at the end of treatment. Shirvani [14] used a pain-level grading curve, indicating the starting level (maximum pain) for a common goal (no pain), so as a primary endpoint we will only evaluate of wound healing end-treatment and pain at mid-treatment; the combined analysis of outcomes related to non-steroidal anti-inflammatory drugs use (NSAIDs), antibiotic use, diet, patient satisfaction. The secondary endpoints evaluate results of the wound healing and pain scores by time in subgroups (start, mid-treatment, and end of treatment).

### 2.5. Quality Assessment

Quality assessment of RCTs was performed by two independent authors (F.B. and S.B.) using the Cochrane Collaboration tool (RoB-2) [15] to assess the risk of bias in randomized trials, where studies are classified as high, low, or unclear risk of bias in 5 domains: selection, performance, detection, attrition, and reporting biases. Disagreements were resolved via consensus after discussing the reasons for the divergence. 

### 2.6. Statistical Analysis

This systematic review and meta-analysis were conducted following the recommendations of the Cochrane Collaboration and the guidelines of the preferred reporting items for systematic reviews and meta-analyses (PRISMA) statement [13]. The mean difference (MD), standardized mean difference (SMD), and odds ratio (ORR) with 95% confidence intervals (CI) was used to compare treatment effects with continuous outcomes. Heterogeneity was examined with the Cochran Q test, *I*^2^ statistic, and visual inspection of the forest plot, and was considered significant if the *p*-value was less than 0.10, the *I*^2^ statistic exceeded 25%, or the visual inspection of the forest plot indicated heterogeneity in the effect size. However, the result of this review did not show visual homogeneity, *I*^2^ > 25%, and *p* < 0.10, suggesting heterogeneity; thus, a random-effects model was used. The statistical analysis was performed using Review Manager 5.3 (Cochrane Centre, The Cochrane Collaboration, Copenhaga, Denmark), and Open Meta-Analyst (http://www.cebm.brown.edu/openmeta/ (accessed on 17 October 2023)) [16] was also used for the statistical analysis.

## 3. Results

### 3.1. Selection and Characteristics

**Figure 1:**
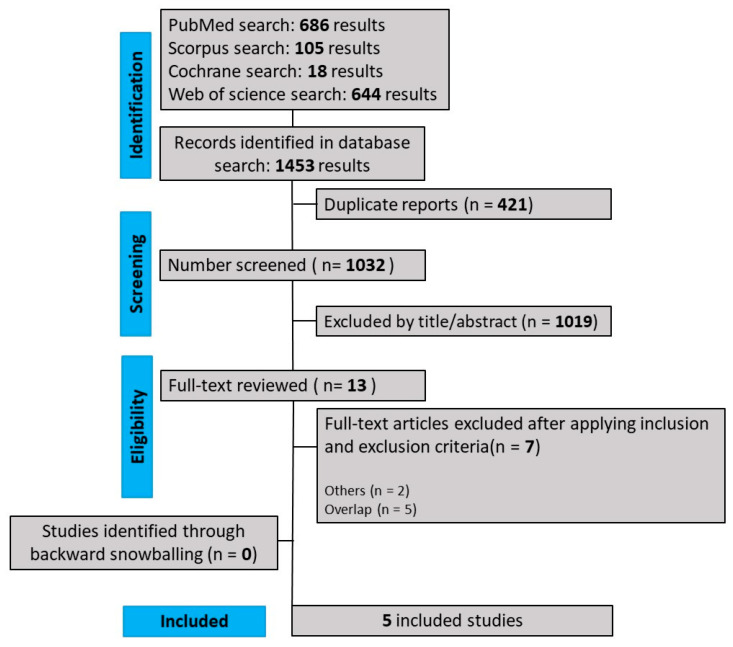
PRISMA flow diagram of study screening.

As detailed in Figure 1, our comprehensive search yielded 1453 results, of which 421 were duplicate records, and 1019 articles were deemed unrelated based on title or abstract review and were excluded. The remaining seven articles were carefully selected and, after evaluating inclusion and exclusion criteria, 5 RCTs [1,14,17,18,19] were included in this systematic review and meta-analysis. The reasons for exclusion of studies were: overlapping studies (*n* = 5) and others (*n* = 2), comprising a study published in another language and a study with measurements (mean/standard deviation) different from those used in this meta-analysis. The main characteristics of individual studies are presented in the Table 1.

### 3.2. Demographic and Clinical Data

After a comprehensive analysis of individual studies, all 353 patients were included. As shown in Table 1, the patients were women undergoing obstetric surgical wounds (cesarean section or episiotomy), with a mean age of 27.90 ± 5.39 years, and shared similar clinical and demographic characteristics at the beginning of the study for both treatment groups. Of these, 177 (50.1%) patients were randomized to receive honey treatment, while the remaining patients received placebo treatment (49.8%).

### 3.3. Combined Analysis of Results and Subgroup Analysis

#### 3.3.1. Wound Healing

Three studies [17,18,19] evaluated wound healing and used the mean and standard deviation to report the data; we chose to evaluate healing only at the end of treatment, between Days 10 and 14, when the wound is usually already completely closed (re-epithelialization ≥ 95%). The data showed no statistical significance between the honey and placebo groups: MD −0.34 [95% CI −1.13, 0.44]; *p* = 0.39; *I*^2^ = 74%, Figure S1.

#### 3.3.2. Pain Intensity Level 

**Figure 2:**
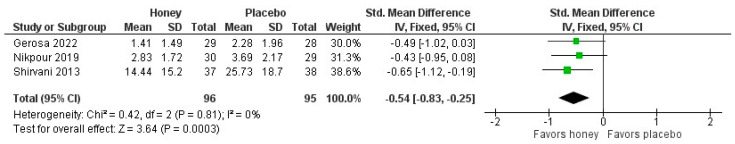
Pain intensity level [1,14,19].

Of the four studies [1,14,17,19] that evaluated the pain intensity during honey treatment of obstetric wounds in the postpartum/cesarean period, three studies [1,14,19] were included in the meta-analysis to evaluate the pain level of obstetric wounds in postpartum women at mid-treatment (Figure 2), showing a significant difference between groups, favoring the beneficial effects of honey in this phase of treatment (SMD −0.54 [95% CI 0.83 to 0.25], *p =* 0.003; *I*^2^ = 0%).

The combined analysis of outcomes related to analgesic use, antibiotic use, diet, patient satisfaction, and study complications was summarized and is presented in Table S1. The five studies [1,14,17,18,19] provided data on combined outcomes. In the Shirvani study [14], patients received 50 mg of diclofenac in the first 24 h and 25 to 50 mg of intramuscular pethidine if pain persisted. NSAIDs were prescribed in the following days as needed and were used by only 11.5% of patients in the intervention group (*p* = 0.02) at the midpoint of treatment. In Shirvani’s study [14], wound redness *(p* = 0.001) and local temperature elevation (*p* = 0.003) were significantly less frequent in the honey group. Regarding wound complications, there were fewer lesions, burning, and itching in the intervention group, but this difference was not significant (*p* = 0.13). Urinary burning was evaluated by Gerosa [1] and no significant results were found. Although the use of honey did not significantly reduce the burning sensation during urination, the women in the control group experienced a significant worsening of the sensation in the presence of abrasions *(p =* 0.03), which supports the hypothesis that honey could help reduce urinary discomfort. The patients in Nikpour, 2019, and Heidari’s study [17,19] received prophylactic intravenous cefazolin after delivery and cephalexin for 7–8 days as antibiotic therapy, along with the option of taking mefenamic acid 25 mg as needed for pain control. 

Gerosa [1] assessed satisfaction only in the honey group (unlike the other authors) using a five-point Likert scale (not at all satisfied, not very satisfied, somewhat satisfied, satisfied, and very satisfied), showing satisfaction in 93% of patients. All patients [1,14,17,18,19] received detailed instructions on wound and suture care, personal hygiene, and nutrition [1,14,17,18] (daily consumption of milk, meat, fruits, vegetables, and grains).

**Figure 3:**
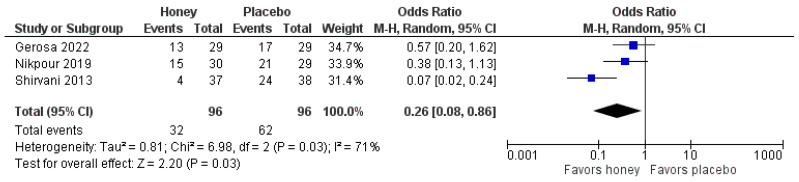
Use of NSAIDs for pain control [1,14,19].

A meta-analysis was performed on the use of NSAIDs for pain control, which showed a significant reduction in the honey-treated group, demonstrating its efficacy (ORR 0.26 [95% CI 0.08, 0.86], *p* = 0.03; *I*^2^ = 71%, Figure 3).

### 3.4. Secondary Outcomes 

The results of the wound healing and pain scores by time in subgroups (start, mid-treatment, and end of treatment) were summarized and presented in Table S2, the results were divided into two groups, and only mid-treatment pain showed statistical significance (*p* < 0.0003). Personal satisfaction was analyzed by a single-arm meta-analysis that demonstrated 81% of patients believed the use of honey to be beneficial and reported high satisfaction (ORR 0.81 [95% CI 0.65, 0.98]; *I*^2^ = 82.16%, Figure S2). 

### 3.5. Quality Assessment

**Figure 4:**
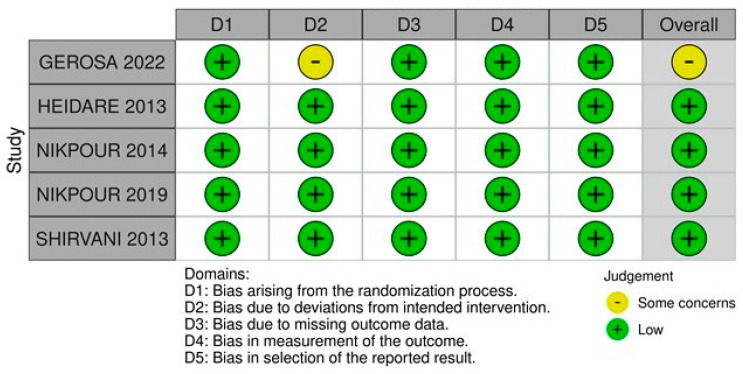
Risk of bias domains [1,14,17,18,19].

Figure 4 summarizes the individual assessment of each RCT included in the meta-analysis. Four studies [14,17,18,19] were classified as having a low risk of bias, while one [1] was classified as having some concerns due to limitations in the randomization process, expressed in the assessment of two domains. 

### 3.6. Sensitivity Analysis

No study was excluded due to methodological heterogeneity, although there was a high degree of statistical heterogeneity (*I*^2^ > 25%) in the results related to wound healing (end of treatment), a sensitivity analysis (Figure S3) was performed systematically excluding each study due to the level of heterogeneity observed (*I*^2^ = 74%), which decreased to 0% after the withdrawal of the study by Nikpour, 2014 [18].

In the results relating to the administration of analgesics, a sensitivity analysis was performed (Figure S4) in which each study was systematically excluded because of the level of heterogeneity observed (*I*^2^ = 71%), which decreased to 0% after the Shirvani study [14] was withdrawn, and in the analysis of the outcome of personal satisfaction (Figure S5), which was also responsible for the increase in heterogeneity.

## 4. Discussion 

The main objective of this study was to demonstrate the benefit of using honey compared to standard treatment with a placebo group for wounds in obstetric procedures (cesarean section, episiotomy, and/or lacerations). This study did not show a significant difference between the intervention group (honey) versus placebo in relation to wound healing; however, in the meta-analysis, the level of pain in postpartum women was evaluated in three measurements, and we observed that at the midpoint of treatment, there was a significant difference between the honey and placebo groups (*p* = 0.003), favoring the use of honey in reducing pain intensity. There was also a reduction in the use of pain medication (*p* = 0.03) and an increase in personal satisfaction in women who underwent the intervention (ORR 0.81), mainly supported by a reduction in complications in various parameters.

The applicability of honey for therapeutic purposes has been used by humanity for thousands of years due to its antimicrobial, anti-inflammatory, and healing properties, with the latter being less well known because their mechanisms have not been demonstrated with precision [20,21]. However, a growing number of clinical trials have been developed and have shown that when applied directly to a wound, there is a reduction in inflammation associated with a potential acceleration in the healing process [8,10,22]. 

Similarly, four previous studies [17,18,23,24] reported that honey had significant effects on wound healing, and one study [14] on pain intensity, while three studies [1,19,23] did not show positive effects of honey cream on pain intensity after cesarean section, and two [17,19] did not show a difference in wound healing. Studies have demonstrated a slight benefit of honey when related to the sensation of urinary burning in lacerations, although honey did not demonstrate overall pain control characteristics, as patients in the control group reported a greater sensation of urinary burning in the presence of abrasions [1,23].

Our results support that honey is effective in reducing pain and is associated with a decrease in the use of pain control medication (*p* = 0.03). Similarly, the use of ylang oil and lemon oil were associated with a decrease in mean pain scores (*p* = 0.005) in women in labor [25], and chamomile oil was also associated with a decrease in labor pain intensity and anxiety, compared to the control group (*p* < 0.001) [26].

The evaluation of episiotomy healing by comparing the total score of five variables from the REEDA scale showed significant differences in relation to wound secretion, especially on the seventh day postpartum (*p* = 0.011), demonstrating a reduction in wound secretions, as well as an increase in episiotomy healing in the honey cream group, which was significantly higher than in the placebo group [23]. A previous study observed that limitations may be related to individual differences among women (ability to regenerate wounds, mobility, and different perineal tissues), which may have caused deviations in wound healing and pain intensity [19].

Nikpour et al. [18] showed that the REEDA score was significantly different between the groups on Days 7 and 14 of treatment; his repeated measures tests showed a significant result over time (*p* < 0.001) and also a significant difference between the two groups in wound healing (*p* = 0.010); different from the results obtained in this meta-analysis, which may mean an insufficient number of participants to evaluate the outcome, a Some of the mentioned studies support the findings of the current study; Heidari et al. [17] showed that using honey for cesarean section scar and wounds did not have any effects on the healing process or pain relief compared to placebo. When studying the participants’ satisfaction with the treatment received, Nikpour et al. [18] demonstrated that in the honey group, the satisfaction rate for wound-healing status was significantly different from the other study groups (*p* < 0.001), with 86% of the participants scoring very high satisfaction compared to the placebo group (26%).

In the meta-analysis of wound healing at the “end of treatment”, a high heterogeneity of 74% was observed due to a study by Nikpour, 2014 [18], probably due to the fact that the authors defined the final healing time as 14 days, which corresponds to a time interval that is 4 days longer compared to the other studies [17,19]. With regard to the outcomes related to the administration of analgesics and personal satisfaction with the use of honey, a sensitivity analysis was performed, which observed a decrease in heterogeneity after the withdrawal of the Shirvani study [14], and it is believed that the type of wound caused the differentiation in the outcome related to the use of analgesics. Satisfaction was subjective, based on personal opinions of what each patient felt comfortable with as medicine in a critical period; conflicting personal opinions could contribute to a possible increase in heterogeneity between studies.

The type of honey used should also be considered, as the properties may vary from one type to another. Nikpour [18,19], and Shirvani et al. [14] used natural honey from the northern region of Iran made from coriander and hawthorn flowers. However, Heidari et al. [17] used honey from the Iranian plant Astragalus gossypinus for Caesarean wounds. Gerosa studied [1] Medihoney^®^ (Brisbane, QLD, Australia), a wound gel consisting of 80% Manuka honey and 20% wax, known to be antibacterial against pathogens such as Staphylococcus aureus and Escherichia coli, for the treatment of perineal lacerations; as these wounds are generally not sutured, their healing time may be longer than other obstetric wounds. 

An important point to be observed is that due to the variable nutritional behavior of bees, they collect substances from different plants, which means that the composition of the produced honey can vary its pharmacological activity, leading to different effects on wound healing [20]. According to the analyzed studies, the efficiency of various honey species can be verified at different levels in wound healing. Heidari et al. [17] showed that honey from A. gossypinus (white astragalus) prepared from herb sources in the Qamsar region seems to be more effective in wound healing. Another study points out that Manuka honey is the flower honey that seems to provide the best medicinal results and is widely used [22].

The patients who used honey were instructed to apply it to the traumatized area after hygiene, avoiding intrusion into the vaginal canal, at least twice a day for the first 5 days; in Nikpour’s study [19], the use of honey was extended to 10 days, and Shirvani and Nikpour, 2014, [14,18] recommended the use of honey gel on the sutures twice a day (12 ± 2 h) for 14 days, without the presence of a dressing on the wound, for 15 min. Heidari et al. [17] told the mothers to use honey twice a day for up to 16 consecutive days.

## 5. Limitations

The strength of this meta-analysis was the use of only double-blind randomized controlled trials. The sample size of the population is not a limitation due to the feasibility of the hypothesis and the rarity of treatment for these individuals in the field of obstetrics. Finally, the geographical location, the origin of the patients and the time of evaluation in each study may inadvertently be a biasing factor that contributes to the increased heterogeneity of the results. However, it is believed that the different types of wounds are not an important limitation in pain control because these women shared the same state of uncomplicated physiologic puerperium and were assessed from the same starting point (maximum pain) and end point (no pain). Regarding the healing results, the difference in the wounds and the type of honey in the sample could be an important factor in differentiating the results of the ability to re-epithelialize or regenerate the skin, and more randomized studies are needed in this area. Therefore, new studies are recommended to evaluate the effects of honey in obstetric wound healing. 

## 6. Conclusions

Evidence supports the use of honey in obstetric wounds for medium-term pain control, reduced analgesia and complication rates, and increased patient satisfaction. However, the main results suggest that there is no significant difference in the healing of obstetric wounds, supporting the conclusions of some individual studies and disagreeing with the evidence presented by others. Due to the combination of different wound types and sample sizes, larger randomized controlled trials with standardized time periods and data collection scales are crucial for a more accurate comparison. In addition, these new studies should be replicated to determine whether the results are maintained in different ethnic groups. The treatment was well tolerated with a good safety in all the studies and is indicated for pain control, reduced use of analgesics, reduced complications, and greater patient satisfaction.

## Figures and Tables

**Table 1:** Demographic and clinical data.

	Gerosa, 2022 [1]		Nikpour, 2014 [18]		Nikpour, 2019 [19]		Heidari, 2013 [17]		Shirvani, 2013 [14]	
	Placebo	Honey	Placebo	Honey	Placebo	Honey	Placebo	Honey	Placebo	Honey
Location; Period	Maternity hospital of the University Hospital of Geneva (2018–2019)		North of Iran		Shahid Yahyanejad hospital, Babol, Iran (2014–2016)		Ayatollah Taleghani Hospital in Arak (2008–2010)		Imam Ali Hospital (in the North of Iran) (2010–2011)	
Study design	RCT		RCT		RCT		RCT		RCT	
Honey used	Medihoney^® a^		Coriander and Goat’s-thorn flowers honey		Coriander and Goat´s-thorn flowers honey		Astragalus gossypinus honey		Coriander and Goat´s-thorn flowers honey	
Type of wound	Perineal laceration		Cesarean section		Episiotomy		Cesarean section		Cesarean section	
Sample size	31	29	38	37	29	30	40	44	38	37
Age (years): mean ± SD	33.80 ± 5.04	33.31 ± 4.31	26.57 ± 4.88	27.70 ± 4.97	24.39 ± 4.21	25.12 ± 3.72	27.38 ± 5.19	27.48 ± 4.31	26.57 ± 4.88	27.77 ± 4.97
Education *n* (%): Primary school	2/31 (6.45) ^b^	3/29 (10.34) ^b^	3 (50)	3 (50)	9 (31.0)	12 (40.0)	NA	NA	18 (21.1)	11 (29.7)
High school	7/31 (22.58)	4/29 (13.79)	25 (52)	23 (48)	14 (46.7)	14 (46.7)	NA	NA	20 (52.6)	15 (40.5)
University	7/31 (22.58)	4/29 (13.79)	10 (48)	11 (52)	6 (20.7)	4 (13.3)	NA	NA	10 (26.3)	11 (29.7)
Mother’s occupation Housewife	NA	NA	31 (53)	28 (48)	24 (82.8)	25 (83.3)	NA	NA	30 (78.9)	28 (75.5)
Employed	NA	NA	At home 7(44)	At home 9(56)	5 (17.2)	5 (16.7)	NA	NA	8 (21.1)	9 (24.3)
Mean neonate weight (SD)	3285 ± 570	3357 ± 350	NA	NA	3243 ± 29.65	3239 ± 35.69	3247.86 ± 397.86	3220.68 ± 406.89	NA	NA

Data presented as the mean ± SD or *n* (%); NA = Not available; ^a^ Medihoney^®^ (Brisbane, QLD, Australia) wound gel, which is made of 80% Manuka honey and 20% wax; ^b^ and Secondary school: placebo—4/31 (12.90), and honey—2/29 (6.90); Factual data extracted from each study were used in this meta-analysis for the evaluation of the main outcomes. Regarding wound healing, the mean and SD from the [17,18,19] studies were used in the REEDA score calculation. For pain assessment, the calculation was based on the mean and SD [1,14,17,19] using the VAS scale.

## Data Availability

Not applicable.

## Supplementary Materials

The following supporting information can be downloaded at: https://www.mdpi.com/article/10.3390/nu16020185/s1, Figure S1—Wound healing; (MD 0.34; 95% CI −1.13 to 0.44; *p* = 0.39); Table S1—Combined analysis of outcomes; Figure S2—Proportion of personal satisfaction; (OR 0.81; 95% CI 0.61, 0.98; *I*^2^ = 82.16); Table S2—Statistical analysis of the outcomes of interest; Figure S3—Wound healing one out analysis; (MD 0.04; 95% CI −0.45 to 0.52; *p* = 0.89); Figure S4—Use of NSAIDs for pain control one out analysis; (OR 0.47; 95% CI 0.22, 1.00; *p* < 0.005); Figure S5—Personal satisfaction one out analysis; (OR 0.81; 95% CI 0.65, 0.98).

## Abbreviations

WHO: World Health Organization; RCT, Randomized Clinical; PRISMA, Preferred Reporting Items for Systematic Reviews and Meta-Analysis; VAS, Visual Analog Scale; MD, Mean Difference; SMD, Standard Mean Difference; NSAIDs, non-steroidal anti-inflammatory drugs; REEDA scale—Redness, Edema, Ecchymosis, Discharge and Approximation of wound edges; ORR, Odds ratio.
