# Supplementary material for: The Use of Honey for Cicatrization and Pain Control of Obstetric Wounds: A Systematic Review and Meta-Analysis of Randomized Controlled Trials
Source: Nutrients. 2024 Jan 5;16(2):185. doi: 10.3390/nu16020185 (PMC10820177; doi:10.3390/nu16020185)
Supplement: Supplementary file 1 [file nutrients-16-00185-s001.zip › nutrients-2670796-supplementary.pdf]

Supplementary Data

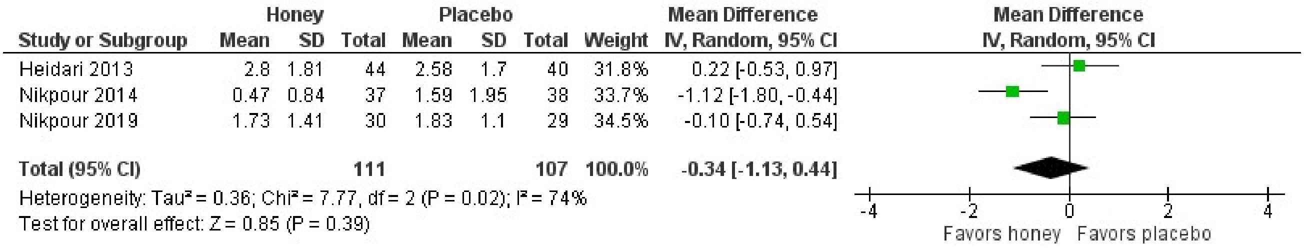

Figure S1 - Wound Healing; (MD 0.34; 95% CI -1.13 to 0.44; *p*=0.39)

Table S1 - Combined analysis of outcomes

Study /year	Pain relief drugs (%)	Antibiotic (%)	Diet*	Satisfaction (%) and p-value	Wound complications[a] (comparison)
Gerosa 2022					
Mel	46.43 %	NA	diet	93%	Mean VAS urinary burning
Placebo	54.84%	NA	advice	NA	($p = 0.31$)
Heidari 2013					
Mel	NA	NA	diet	Not significant	Not significant difference
Placebo	NA	NA	advice	difference	
Nikpour 2014					
Mel	NA	89%	diet advice	86% (p<0,001)	Rednees ($p< 0.003$) Edema ($p = 0.010$) Ecchymosed ($p = 0.006$)
Placebo	NA	94%		26%	
Nikpour 2019					
Mel	50%	73.3%	NA	NA	Tingling and itching ($p =$ 0.921)
Placebo	72.4%	82.8%	NA	NA	
Shirvani 2013					
Mel	11,5%[b]	NA	diet advice	61% (p<0,001)	Burn ($p = 0.13$) Pruritis ($p = 0.13$) Redness ($p = 0.001$) Warming ($p = 0.003$)
Placebo	62,6% [b]	NA		17,01%	

NA, not available; * milk, meat, fruits, vegetables and cereals intake; [a] always evaluated on the last day of treatment; [b] first 10 days.

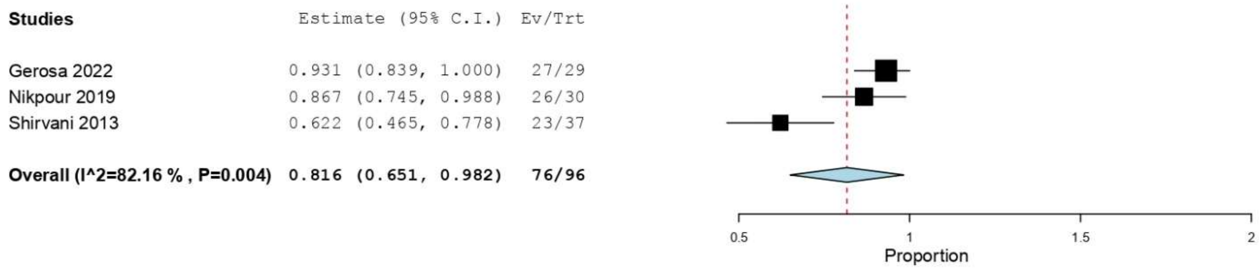

Figure S2 – Proportion of Personal satisfaction; (OR 0.81; 95% CI 0.61, 0.98; I^2=82.16)

Table S2 - Statistical analysis of the outcomes of interest.

OUTCOMES	STUDIES	Nª OF PATIENTS	MD OR SMD	95% IC	P-VALUE	HETEROGENEITY CHI²	DF	P-VALUE	I²(%)
Wound healing (middle of treatment)	2	134	- 0.84	- 2.23, 0.55	0.24	4.21	1	0.04	76%
Woudn healing (end of treatment)	3	218	- 0.34	- 1.13, 0.44	0.39	7.77	2	0.02	74%
Pain intensity (24 h)	3	217	0.11	- 0.15, 0.38	0.40	1.09	2	0.58	0%
Pain intensity (middle of treatment)	3	191	- 0.54	- 0.83, - 0.25	0.0003*	0.42	2	0.81	0%
Pain intensity (end of treatment)	3	218	- 0.71	- 1.92, 0.49	0.24	6.94	2	0.03	71%

* Statistically significant. MD, mean difference; SMD, standardized mean difference; CI, confidence interval;

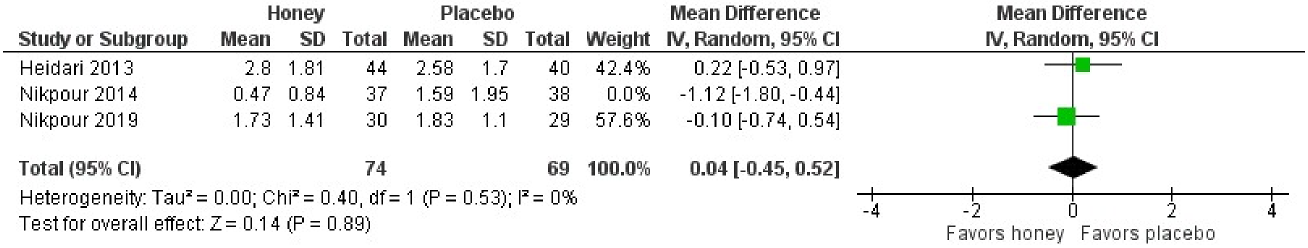

Figure S3 – Wound Healing one out analysis; (MD 0.04; 95% CI -0.45 to 0.52; *p*=0.89)

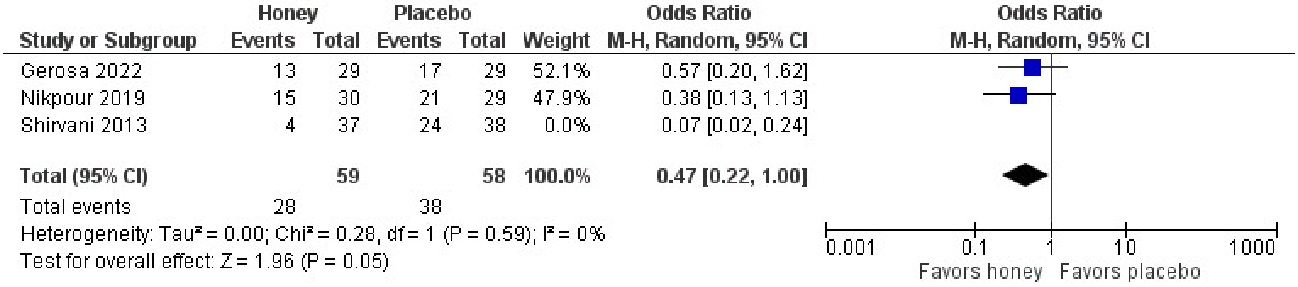

Figure S4 – Use of NSAIDs for pain control one out analysis; (OR 0.47; 95% CI 0.22, 1.00; *p*<0.005)

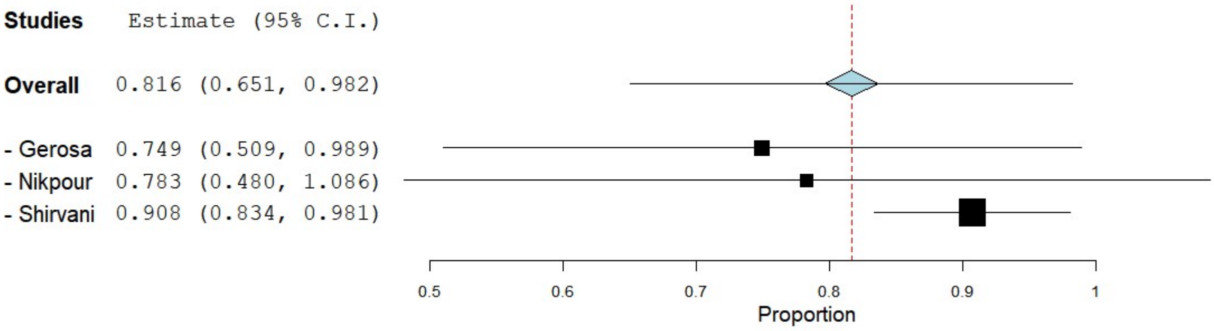

Figure S5 – Personal satisfaction one out analysis; (OR 0.81; 95% CI 0.65, 0.98;)
